# Mucosal-Associated Invariant T Cell Response to Acute Exercise and Exercise Training in Older Obese Women

**DOI:** 10.3390/sports9100133

**Published:** 2021-09-24

**Authors:** Lauren C. Bates, Erik D. Hanson, Michael M. Levitt, Bryan Richie, Elise Erickson, David B. Bartlett, Melody D. Phillips

**Affiliations:** 1 Department of Exercise and Sport Science, University of North Carolina, Chapel Hill, NC 27599, USA; lbates15@live.unc.edu; 2 Human Movement Science Curriculum, University of North Carolina, Chapel Hill, NC 27599, USA; 3 Lineberger Comprehensive Cancer Center, University of North Carolina, Chapel Hill, NC 27599, USA; 4 Department of Kinesiology, Texas Christian University, Fort Worth, TX 76109, USA; mike.levitt@outlook.com (M.M.L.); bryan.richie@tcu.edu (B.R.); eerickson@twu.edu (E.E.); melodydphillips@outlook.com (M.D.P.); 5 Duke Molecular Physiology Institute, Duke University, Durham, NC 27701, USA; david.bartlett@duke.edu; 6 Department of Nutritional Sciences, Faculty of Health and Medical Sciences, University of Surrey, Guildford GU2 7XH, UK

**Keywords:** exercise immunology, aerobic exercise, resistance training, overweight, obesity, post-menopausal, women, mucosal-associated-invariant T-cells

## Abstract

(1) Background: Obesity is a major global public health concern as it is associated with many of the leading causes of preventable deaths. Exercise reduces obesity-induced inflammation; however, it is unknown how exercise training may impact mucosal associated invariant T (MAIT) cells in overweight/obese (OW) post-menopausal women. Therefore, the purpose of this study was to investigate (i) circulating MAIT-cells at rest in OW vs. Lean women, (ii) the response of MAIT-cells to a single bout of combined aerobic and resistance exercise, and (iii) the effects of 12 weeks of exercise training (EX) or educational program (ED) on the MAIT-cell response in OW. (2) Methods: OW completed an acute exercise session or sitting control, underwent 12 weeks of exercise training or received educational materials, and then repeated the exercise session/sitting control. Lean post-menopausal women provided a baseline comparison. (3) Results: OW had lower circulating MAIT-cells at rest than Lean prior to exercise training; however, after training EX displayed improved MAIT-cell frequency. Additionally, prior to training EX did not exhibit MAIT-cell mobilization/egress, however, both improved after training. (4) Conclusions: Reduced MAIT-cell frequency and ability to mobilize/egress were potentially partially rescued in EX after 12 weeks of exercise training; however, further research is needed to elucidate age or obesity-induced attenuations in MAIT-cells.

## 1. Introduction

Obesity is associated with many of the leading causes of preventable deaths (heart disease, diabetes, etc.) and is a major health concern as globally 40% of adult women are overweight (BMI: ≥ 25 kg/m^2^) and 15% are obese (BMI ≥ 30 kg/m^2^) [1]. Many long-term health consequences such as cardiometabolic diseases are associated with the systemic chronic low-grade inflammation and vascular dysfunction caused by obesity [2]. Furthermore, pro-inflammatory immune cells and an increase in the production of pro-inflammatory cytokines are a key characteristic of obesity [3]. Weight loss strategies such as exercise (alone or in combination with nutrition intervention) serve as potential treatment for obesity [4] as it is known to reduce obesity-induced chronic inflammation [5].

Obesity causes systemic physiological dysfunction such as inflammatory-induced lymphoid tissue change (e.g., bone marrow and thymus) and altered distribution of leukocytes subsets, especially towards a proinflammatory phenotype [6]. One type of unconventional T-cell that possesses qualities of innate and adaptive immunity are mucosal-associated invariant T (MAIT) cells. MAIT-cells are identified by the expression of a semi-invariant T-cell receptor (TCR) Vα7.2 and CD161 [7]. Following activation, MAIT-cells have the ability to rapidly produce Th17 cytokines (e.g., IL-17, TNF-α, IFN-γ) while also possessing cytotoxic function [7]. MAIT-cells are reportedly altered in obese populations including lower MAIT-cell counts/frequencies in peripheral blood [8,9,10,11,12], and differing cytokine production (compared to healthy controls) including a reduced (or even lost) ability to produce IFN-γ [13] and an elevated IL-17 production [11]. Furthermore, in an obese population with un-detectable levels of MAIT-cells, after undergoing bariatric surgery MAIT-cells began to recover [14]. Therefore, weight loss strategies may be effective in rescuing obesity-induced declines in MAIT-cell counts and function. 

While not as potent as bariatric surgery for reducing fat mass, exercise has the ability to improve body composition, along with other direct benefits to the immune system including increased T-cell proliferative capacity and lower circulatory levels of inflammatory cytokines [15]. Furthermore, MAIT-cells mobilize following exercise. In healthy young men, maximal acute exercise increased MAIT-cell counts by 2.2-fold and cell frequencies by 0.8%, suggesting a preferential mobilization among T-cell populations [16]. In response to submaximal acute aerobic exercise, MAIT-cell counts increased by 92% and cell frequency by 1.0% along with an increased proportion of mitogen stimulated MAIT-cells expressing TNF-α was reported, indicating increased sensitivity to MAIT-cell stimulation [17]. MAIT-cells have displayed an intensity dependent numerical mobilization similar to conventional CD8+ T-cells [18]. Exercise training has been shown to partially rescue acute exercise-induced MAIT-cell mobilization in older breast cancer survivors [19]. Although the population of breast cancer survivors was not overweight, breast cancer and its treatment results in similar proinflammatory and reduced immune function as obesity. MAIT-cells are responsive to acute exercise in healthy populations, can be partially rescued by exercise training in a clinical population, and are responsive to weight loss strategies such as bariatric surgery. However, the effects of acute exercise and exercise training on MAIT-cell counts and functions in obese and female populations remain largely unknown. The purpose of this preliminary study is to investigate (i) circulating MAIT-cells at rest in overweight/obese (OW) and lean post-menopausal women, (ii) the response to a single combined (aerobic and resistance training) acute exercise bout, and (iii) the effects of a 12-week combined resistance and aerobic exercise training (EX) or educational (ED) program on the acute MAIT-cell response in OW post-menopausal women.

## 2. Materials and Methods

### 2.1. Participants 

Sixteen inactive, overweight/obese (OW) and eight moderately active lean (reference controls) post-menopausal women participated in this study. Inactive was defined as not participating in exercise regularly for the previous six months (more than one moderate exercise bout per week). Post-menopausal was defined as not experiencing a menstrual cycle for two or more years (due to natural or surgical menopause). Exclusionary criteria included chronic inflammatory diseases or autoimmune disorders (e.g., Addison’s disease, Graves’ disease, multiple sclerosis, systemic lupus erythematosus, rheumatoid arthritis and psoriaticarthritis, HIV/AIDS, acute or chronic infection, previous myocardial infarction, peripheral artery disease (PAD), Type 1 or 2 diabetes mellitus, chronic respiratory condition, blood disorders), oral steroid or statins use, and those unable to exercise due to an injury or long-term illness.

### 2.2. Experimental Protocol 

Upon approval by institutional review board at Texas Christian University, participants were recruited via newspaper ads, flyers, electronic postings, and word-of-mouth from university campus and surrounding community. Participants then provided written informed consent, a medical history form and physical activity questionnaire and underwent a medical screening by a personal physician and our study physician prior to participation in the study to insure there were no contraindications to exercise. 

### 2.3. Experimental Design 

**Figure 1:**
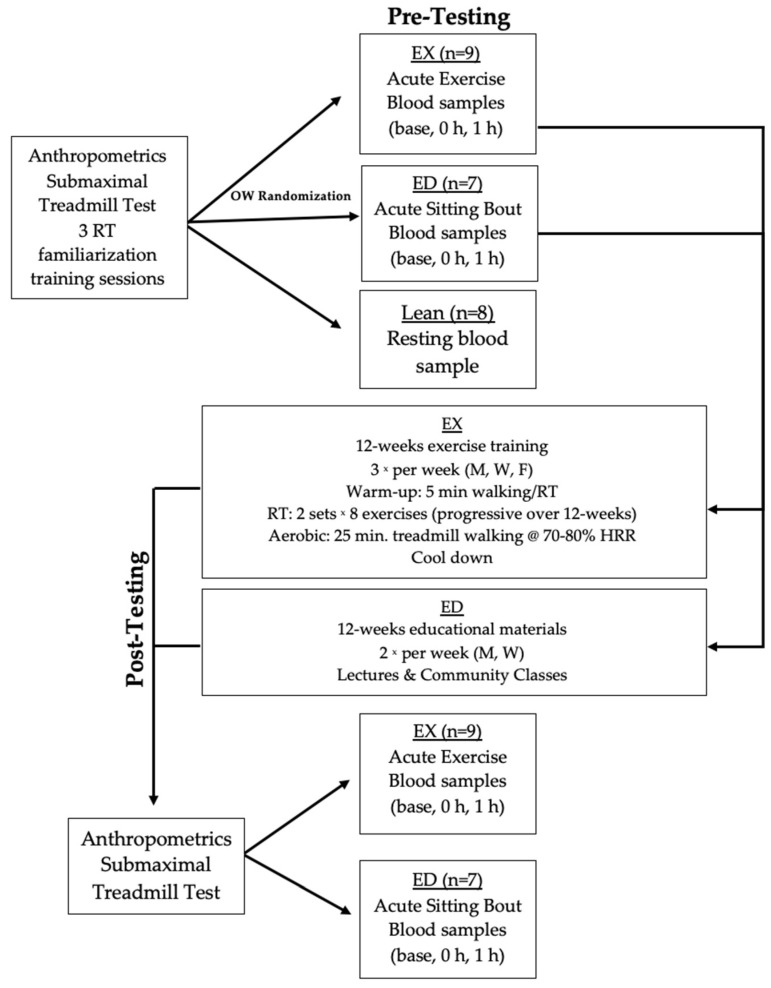
Experimental timeline including pre-training acute exercise testing, 12 weeks of exercise training (EX) or educational materials (ED) exposure, and then post-training acute exercise testing. Abbreviations: Lean, lean controls; OW, overweight/obese participants; RT, resistance training; M, Monday; W, Wednesday; F, Friday; base, baseline; 0 h, immediately post-exercise; 1 h, 1 h after exercise.

This analysis was part of a larger study [20]. To examine the MAIT-cell response to acute exercise, OW were randomized into an exercise (EX) group and a non-exercise, education (ED) reference group. Lean provided a resting blood sample to serve as a reference control when compared to OW. The timeline is provided in Figure 1 and details about the acute exercise bout and exercise training are provided below. 

### 2.4. Acute Exercise Testing 

On the first day, OW participants reported to the Exercise Physiology Lab at Texas Christian University and anthropometrics were measured including height, weight, and BMI. Participants then underwent a submaximal (85% of heart rate reserve) treadmill test (used to estimate VO_2_ max via the linear extrapolation method). Participants then completed 3 days of familiarization/acclimation to resistance exercises including one day where they were taught proper lifting techniques (8 repetition maximum (RM) was assessed for each of the resistance exercises), a second day where participants completed three sets of each exercise at 50% of their estimated 1RM, and a third day where participant’s 8RM for all exercises was reassessed. Resistance exercises included: leg extension and flexion, leg press, hip adduction and abduction, chest press, seated row and latissimus dorsi muscle pulldown. 

Both before (Pre) and after (Post) 12 weeks of exercise training, OW participants reported to the lab following a 10-h overnight fast and assumed a supine position for 15-min prior to a resting heart rate, supine resting blood pressure (BP) measurements as a safety precaution. An intravenous catheter was inserted into the antecubital vein of the forearm for repeated blood sampling, and a baseline resting sample was taken. EX completed 2 sets of 8 different resistance training exercises and 25 min of walking at 70–80% heart rate reserve. Blood draws occurred immediately following exercise (0 h) and then after 1-h of rest (1 h). The ED group completed blood sampling at the same timepoints as EX, however ED sat for the duration of the visit to provide reference as an acute non-exercise control.

### 2.5. Lean Acute Exercise Testing

A sample of convenience of age-matched lean participants (Lean) also reported to the Texas Christian University Exercise Physiology Lab for preliminary paperwork and anthropometrics measurements. Lean underwent a submaximal (85% heart rate reserve) treadmill exercise test, also used to predict VO_2_ max, as described above. At a second visit, Lean arrived at the Exercise Physiology Lab after a 10-h overnight fast and assumed a supine position for 15-min prior to a resting heart rate, blood pressure and a single blood sample via standard venipuncture techniques to serve as a baseline healthy comparison to OW. 

### 2.6. Exercise Training

EX participants completed 12 weeks of combined exercise training (aerobic and resistance) three times per week on non-consecutive days (i.e., Monday, Wednesday, and Friday). Each session included a 5-min walking warm-up, resistance training, aerobic training, and a cool-down period. Participants first completed resistance exercises at 75–100% of measured 8RM, consisting of an initial set of eight repetitions followed by a second set performed to ‘‘momentary muscular failure.’’ The exercise training was progressive as the participants’ number of repetitions were recorded daily and the resistance for each exercise was adjusted using a standardized method as strength improved. For example, if participants were capable of performing 12 or more repetitions on 3 consecutive workouts, exercise load was increased by 10 lbs. (4.54 kg) in lower body exercises and 5 lbs. (2.27 kg) in upper body exercises. Aerobic training consisted of treadmill walking for 25 min at 70–80% of heart rate reserve. 

All exercise sessions were closely monitored by trained exercise technicians. All EX participants completed at least 36 exercise sessions. In instances where a participant missed a session(s) due to illness or travel, the session(s) was made up upon return to training. If the missed session(s) occurred during the last four weeks of training, an additional three training sessions (one week) were added to ensure that any fitness gains thus far were not lost prior to the post-training experimental trial. A similar process was used for ED participants and their education sessions. In the larger trial, exercise adherence was 90%. The ED group met in a group for education sessions two times per week including guest lectures, community classes, crafts and games, and personal safety to control for diurnal and seasonal variations. ED meetings also provided social interaction between participants and were instructed to not modify their lifestyle (e.g., diet and exercise) throughout the 12 weeks of participation. 

### 2.7. Blood Analysis

Blood was collected into chilled ethylenediaminetetraacetic acid (EDTA) tubes following manufacturer’s instructions (Invitrogen, Carlsbad, CA, USA) and analyzed using ActDiff 5 hematology analyzer (Beckman Coulter, Brea, CA, USA). PBMCs were cryopreserved utilizing methodology previously described [19]. At the end of the study, PBMCs were thawed in a 37 °C warm water bath. Then 1 mL of room temperature RPMI Complete Media (10% FBS, 1% Penicillin-Streptomycin in RPMI) was added in dropwise manner to the PBMCs. Cells were counted using an automated cell counter (TC-20 Bio-Rad, Hercules, CA, USA) that was verified via hemocytometer. Cell viability was verified using Trypan Blue (Bio Rad, Hercules, CA, USA). PBMCs were allowed to rest for 2 h at 37 °C and 5% CO_2_ (confirmed by pilot work). Cell viability was again assessed and then cells were stimulated with 2 ng/mL phorbol 12-myristate 13-acetate (PMA) and 1 ng/mL Ionomycin for 4 h at 37 °C and 5% CO_2_, as used previously [19]. 

**Figure 2:**
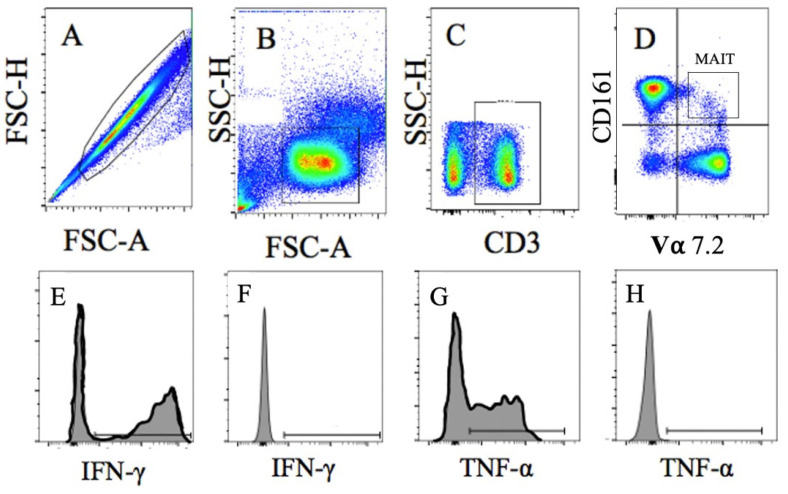
Representative Image of MAIT Cell Gating Strategy. Cells were gated as (**A**). Singlets, then as (**B**). lymphocytes, followed by (**C**). CD3^+^T Cells, and then (**D**). CD161/Vα7.2 T Cells (MAIT-cells). Intracellular MAIT-cell cytokine levels were determined following stimulation for (**E**). IFN-λ positive and (**F**). negative control and (**G**). TNF-α positive and (**H**). negative control.

Cells were phenotyped using immunofluorescence labeling of cell surfaces with mouse anti-human monoclonal antibodies prior to stimulation, as previously described [7]. PBMCs were also stimulated with 2 ng/mL phorbol 12-myristate 13-acetate (PMA) and 1 ng/mL ionomycin or vehicle controls for 4 h at 37 °C and 5% CO_2_ to induce intracellular cytokine production [7] that was stained using mouse anti-human monoclonal antibodies in fixation and permeabilization buffer (BD Biosciences, Franklin Lakes, NC, USA). Cells were washed to remove excess antibody and suspended in 300 μL of cell staining buffer for flow cytometry analysis. Cells were analyzed using flow cytometry (LSRII with Diva Software, BD Biosciences, NC, USA) and FlowJo 8 CE software (Ashland, OR, USA), with the gating strategy shown in Figure 2. 

### 2.8. Statistical Analysis

Data were collected and analyzed with SPSS Statistics version 25.0 (SPSS, IN., Durham, NC, USA) and with Jamovi Statistics Version 1.2.27.0 (Jamovi Project Computer Software, 2020). Figures were created in GraphPad Prism version 9 (La Jolla, CA, USA). Investigations that were assessed in this study included: (i) differences between the Lean and OW at rest prior to training (Pre), (ii) differences between the EX and ED in their acute exercise response at Pre, and (iii) differences between the EX and ED in their acute exercise response following training (Post). For Outcome 1, descriptive statistics (mean ± SD) are shown for baseline characteristics and compared across groups (Lean vs. OW) via t-test. For Outcomes 2 and 3, mobilization and egress were analyzed separately using a linear mixed model [19]. The fixed factors were group and time with subjects as a random effect. Mobilization was defined as the change from baseline to 0 h post-exercise while egress was the change from the 0 h to 1 h post-exercise. For Outcome 3, the effect of exercise training, the immune analyses were examined by the calculation of change scores separately for mobilization and egress, with group and training as fixed factors and subjects as a random effect. As this was a pilot study, effect sizes were calculated as Cohen’s D (d) for all outcomes and were used as the primary comparison with 0.2, 0.5 and 0.8 representing small, medium, and large differences, respectively. Probability testing was also included as a reference, where the α level was set at *p* < 0.05 for main effects while all interactions that were *p* < 0.20 were examined further, given the exploratory nature of this analysis. Percent change was calculated as 100 × [(final – original)/(original)] where Pre baseline or ED were the original for comparison purposes.

## 3. Results

### 3.1. Participants

At Pre, the EX + ED groups (OW) and Lean were age-matched (64 (6) yr., Table 1). The Lean reference group had a superior VO_2_ (8.1 (4.2) mL/kg/min, *p* < 0.001, Table 1), lower weight (32.1 (6.7) kg, *p* < 0.001, Table 1) and lower BMI (11.4 (2.9) kg/m^2^, *p* < 0.001, Table 1) than OW. Additionally, height, weight, BMI and VO_2_ testing were similar between EX and ED.

### 3.2. Baseline Comparison-Pre-Training

**Figure 3:**
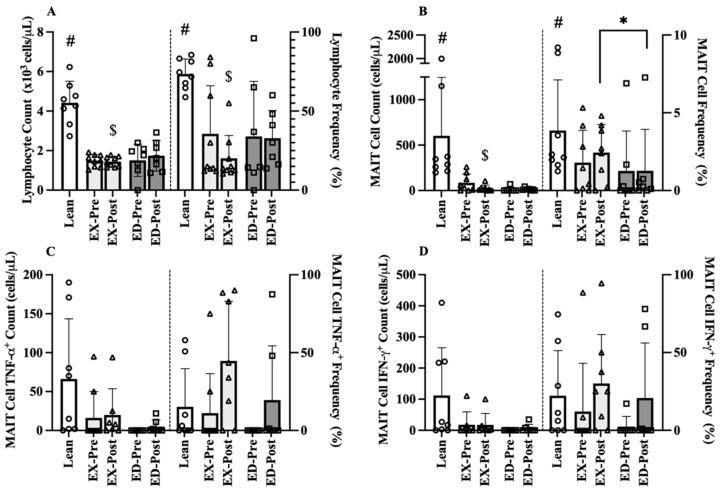
Pre-Training (Pre) and Post-Training (Post) resting immune cell counts and frequencies for Lean, exercise training (EX) and education (ED) groups. (**A**) Lymphocyte counts (left) and frequency (right) (**B**) MAIT-cell counts and frequency, (**C**) TNFα^+^ MAIT-cell counts and frequency, and (**D**) IFNγ^+^ MAIT-cell counts and frequency in age-matched Lean vs. overweight (OW) women at rest before and after training. Data are mean (SD). Abbreviations: MAIT, mucosal associated invariant T-cells; EX, exercise training group; ED, education group; Pre, pre-training; Post, post-training. # *p* < 0.05 vs. OW Pre; $ *p* < 0.05 vs. Lean; * *p* < 0.05 vs. EX Post.

As randomization occurred after the Pre acute exercise, OW participants Pre baseline samples were pooled (n = 16) for comparison to Lean (n = 8). There were large effects between OW and Lean, with OW having 2-fold lower lymphocyte counts (−2.9 × 10^3^/µL cells, 95% CI [−3.1, −2.7], d = 3.27, *p* < 0.001, Figure 3A) and lower lymphocyte frequency (−36.2%, 95% CI [−45.8, −28.0], d = 0.54, *p* = 0.004, Figure 3A) than Lean. Additionally, MAIT-cell counts were 86% lower in OW (−543 × cells, 95% CI [−765, −321], d = 1.18, *p* < 0.001, Figure 3B) along with a lower MAIT-cell frequency (−2.8%, 95% CI [−3.0, −2.0], d = 0.86, *p* = 0.013, Figure 3B) then Lean. Large group effects were observed for MAIT-cell cytokine counts where TNF-α was 86% lower (d = 0.99, *p* = 0.100, Figure 3C) and IFN-γ was 93% lower (−104 × 10^3^/µL cells, 95% CI [−156, −54], d = 0.94, *p* = 0.055, Figure 3D) in OW compared to Lean. Small to medium effects were observed for MAIT-cell frequency of TNF-α (d = 0.40, *p* = 0.337, Figure 3C) and IFN-γ (15.1%, 95% CI [−4.0, 18.0], d = 0.58, *p* = 0.169, Figure 3D) that favored Lean.

### 3.3. Acute Exercise

#### 3.3.1. Acute Hematological Response 

The acute hematological response to exercise was assessed prior to the exercise training or education interventions. A group x time interaction was observed for lymphocytes where EX increased by 52% at 0 h (1 × 10^3^/µL cells, 95% CI [2,3], d = 1.3, *p* < 0.001) while ED (sitting control) did not change (0.1 × 10^3^/µL cells, 95% CI [0.0, 1.0], d = 0.2, *p* = 0.282). Time main effects were observed in all outcomes except hemoglobin (all *p* < 0.05), with large group effects observed for both mobilization and egress across all immunological parameters (Table 2). 

#### 3.3.2. Acute MAIT-Cell Counts and Frequencies 

**Figure 4:**
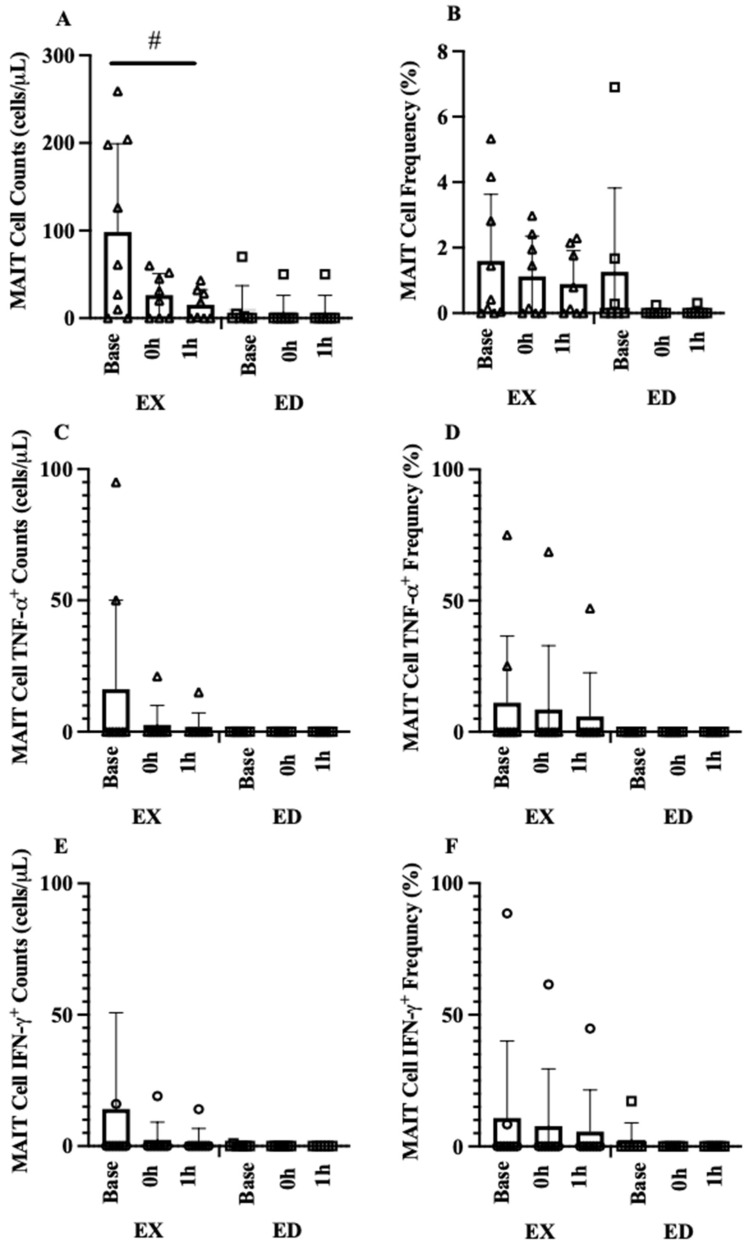
MAIT cell acute exercise response (**A**) MAIT-cell count acute exercise response (EX vs. ED), (**B**) MAIT-cell frequency acute exercise response (EX vs. ED), (**C**) MAIT-cell TNFα^+^ count acute exercise response (EX vs. ED), (**D**) MAIT-cell TNFα^+^ frequency acute exercise response (EX vs. ED), (**E**) MAIT-cell IFNγ^+^ count acute exercise response (EX vs. ED), (**F**) MAIT-cell IFNγ^+^ frequency acute exercise response (EX vs. ED)**.** Data are mean (SD). # *p* < 0.05 vs. ED.

At Pre, there were numerous medium to large group effects sizes, but the overall magnitude of the changes was quite small. Following acute exercise, there was no group × time interaction for MAIT-cell count mobilization. There was a large group main effect where EX MAIT-cell counts were 78.3% higher overall (19 cells, 95% CI [12, 36], d = 1.16, *p* = 0.037, Figure 4A) compared to ED. MAIT-cell frequency was unchanged over time, with only a small overall group effect (d = 0.23, *p* = 0.124, Figure 4B). There were also no interactions for MAIT-cell count mobilization for intracellular cytokines. There were small group effects for TNF-α (d = 0.46, *p* = 0.295, Figure 4C) and IFN-γ (d = 0.38, *p* = 0.381, Figure 4E). MAIT-cell intracellular frequency was low for both cytokines, with no change in the frequency of TNF-α over time (d = 0.07, *p* = 0.359, Figure 4D) while IFN-γ demonstrated small to medium declines (−11.7%, 95% CI [−40.1, 16.7], d = 0.38, *p* = 0.184, Figure 4F). 

### 3.4. Exercise Training

The third component of this study assessed the immune response to acute exercise following 12 weeks of combined aerobic and resistance training for EX and the immune response to acute sitting following 12 weeks of education sessions for ED. 

#### 3.4.1. Baseline Comparison-Post-Training

Following exercise training, Post hematological values at rest (baseline) between the EX and ED groups were compared to the Lean Pre values (Figure 3). Large effect sizes were still observed in EX where lymphocyte counts (−2 × 10^3^/µL cells, 95% CI [−2, −1], d = 3.27, *p* < 0.001) and lymphocyte frequency (−26.5%, 95% CI [−34.0, −19.0], d = 3.56, *p* < 0.001) remained lower than Lean. Large effect sizes were also observed between EX and Lean MAIT-cell counts, where EX counts remained 96% lower counts (584 cells, 95% CI [187, 981], d = 1.30, *p* = 0.005). A medium effect size was observed for MAIT-cell frequency between EX and Lean (1.5%, 95% CI [1.0, 4.0], d = 0.56, *p* = 0.540) whereas a large effect was present between ED and Lean (2.6%, 95% CI [−2.3, 2.9], d = 0.86, *p* = 0.166). A medium effect was observed where EX had higher MAIT-cell counts (11 cells, 95% CI [2, 21], d = 0.43, *p* = 0.165) than ED. A medium effect was observed between EX and ED where EX had higher MAIT-cell frequency (1.2%, 95% CI [0.8, 1.6], d = 0.51, *p* = 0.002). 

#### 3.4.2. MAIT-Cell Counts and Frequencies

**Figure 5:**
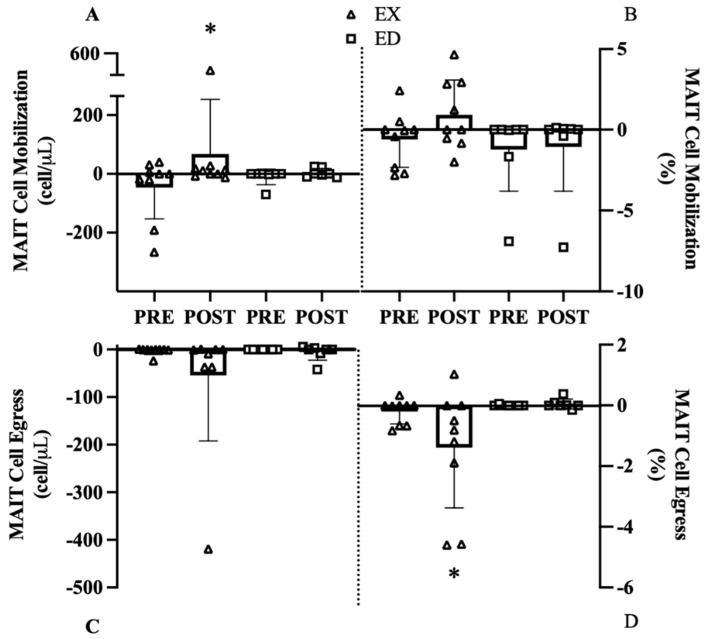
MAIT cell mobilization and egress before (Pre) and after (Post) exercise group (EX) or education group (ED). (**A**) MAIT-cell count mobilization, (**B**) MAIT-cell frequency mobilization, (**C**) MAIT-cell count egress, (**D**) MAIT-cell frequency egress. * *p* < 0.05 vs. Pre.

Mobilization was defined as the change from baseline to 0 h and egress was defined as the change from 0 h to 1 h displayed as change scores before and after exercise training. The MAIT-cell count mobilization interaction was below our exploratory threshold (*p* = 0.176), with a large effect in EX from Pre to Post (114 cells, 95% CI [69, 225], d = 0.99, *p* = 0.028, Figure 5A) but with smaller change in ED (14 cells, 95% CI [5.7, 22.3], d = 0.46, *p* = 0.800). A similar pattern was observed for MAIT-cell frequency where EX exhibited a large change from Pre to Post (1.4%, 95% CI [0.7, 2.1], d = 1.22, *p* = 0.156, Figure 5B) while ED was unchanged, but the interaction was not significant (*p* = 0.395) with no effects of group (*p* = 0.151) or training (*p* = 0.291).

Despite a large effect in EX (d = 1.22, Figure 5C), the MAIT-cell count egress interaction (*p* = 0.395) and main effects of group (*p* = 0.341) or training (*p* = 0.287) were all not significant. The MAIT-cell frequency egress interaction (*p* = 0.077) revealed a strong effect in EX from Pre to Post (−1.2%, 95% CI [−3.0, 0.0], d = 1.50, *p* = 0.014, Figure 5D) with no change in ED (−0.03%, 95% CI [−0.14, 0.07], d = 0.00, *p* = 0.937). 

#### 3.4.3. MAIT-Cell Cytokine Counts and Frequencies

**Figure 6:**
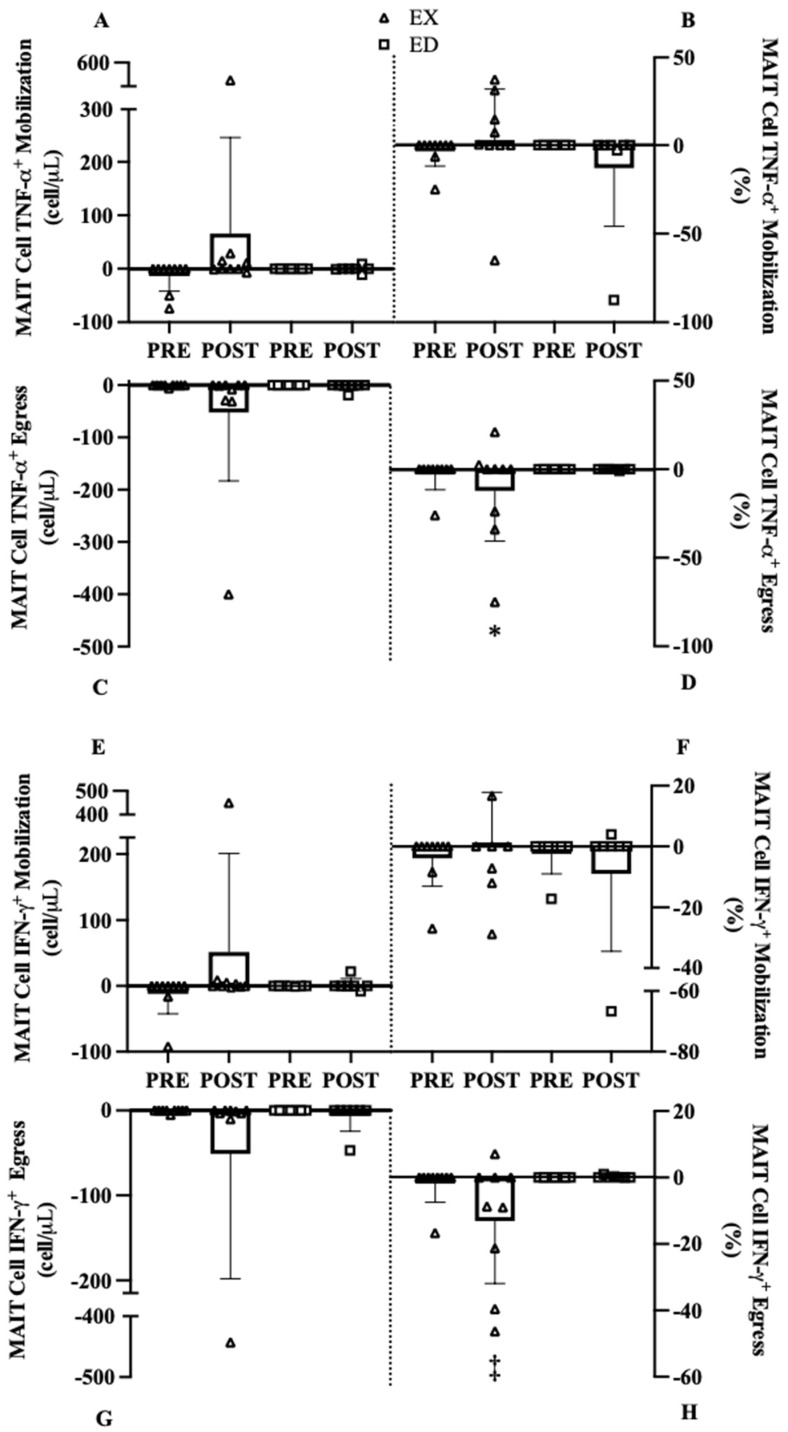
MAIT cell mobilization and egress of MAIT cells expressing TNF-α or IFN-γ before (Pre) and AFTER (Post) exercise training (E^X^) or education controls (ED). (**A**) MAIT-cell TNF-α^+^ count mobilization, (**B**) MAIT-cell TNF-α^+^ frequency mobilization, (**C**) MAIT-cell TNF-α^+^ count egress, (**D**) MAIT-cell TNF-α^+^ frequency egress, (**E**) MAIT-cell IFN-γ+ count mobilization, (**F**) MAIT-cell IFN-γ+ frequency mobilization, (**G**) MAIT-cell IFN-γ+ count egress, (**H)** MAIT-cell IFN-γ+ frequency egress. * *p* < 0.10 vs. Pre, ‡ *p* < 0.05 vs. Pre.

In general, when comparing Pre and Post mobilization and egress, medium to large effect sizes were observed for all cytokine counts and frequencies in EX with minimal change in ED. Despite these large effects, only group x training interactions that met our criterion are discussed. For MAIT-cell TNF-α^+^ counts mobilization, there was no interaction (*p* = 0.259) or main effects of training (*p* = 0.264) or group (*p* = 0.457, Figure 6A). The MAIT-cell TNF-α^+^ frequency mobilization interaction (*p* = 0.182) revealed a large increase in EX (6.4%, 95% CI [−7.6, 20.0], d = 0.80, *p* = 0.492, Figure 6B) where ED decreased mobilization from Pre to Post (−12.9%, 95% CI [−37.3, 11.4], d = 1.02, *p* = 0.232, Figure 6B). For MAIT-cell TNF-α^+^ counts egress, there was no interaction (*p* = 0.339, Figure 6C) or main effects of training (*p* = 0.290) or group (*p* = 0.341). Similarly, no interaction (*p* = 0.428) or main effects of training (*p* = 0.415) or group (*p* = 0.199) were present for MAIT-cell TNF-α^+^ frequency (Figure 6D).

For IFN-γ+ MAIT-cell count mobilization, there was no interaction (*p* = 0.300) or main effects of training (*p* = 0.268) or group (*p* = 0.522, Figure 6E). Similar findings were observed in IFN-γ+ MAIT-cell mobilization for cell frequency where there was no interaction (*p* = 0.223) or main effects of training (*p* = 0.890) or group (*p* = 0.513, Figure 6F). For IFN-γ+ MAIT-cell count egress there was no interaction (*p* = 0.444, Figure 6G) or main effects of training (*p* = 0.321) or group (*p* = 0.443). For IFN-γ+ MAIT-cell frequency egress there was an interaction (*p* = 0.092, Figure 6H) where greater egress occurred in EX (−11.2%, 95% CI [2.1, 20.4], d = 0.81, *p* = 0.018) and ED remained unchanged. 

## 4. Discussion

The purpose of this study was to investigate (i). circulating MAIT-cells at rest in OW and Lean post-menopausal women, (ii). the response to a single combined acute exercise bout (compared to an acute sitting bout), and (iii). the effects of a 12-week combined resistance and aerobic exercise training or educational program on the acute MAIT-cell response in OW post-menopausal women. Following exercise training, resting baseline MAIT-cells in EX remained below levels shown in Lean as hypothesized. At Pre, EX did not demonstrate the anticipated biphasic MAIT-cell acute exercise response. However, at Post greater mobilization and egress were observed. While not all changes reached statistical significance, increases in MAIT-cell number and frequency appear more sensitive to the stress of acute exercise. Therefore, including an acute exercise bout before and after training provides valuable information not captured by only examining MAIT-cell count and frequency at rest. In OW post-menopausal women 12 weeks of exercise training may elicit a partial restoration of the MAIT-cell response; however, more research is needed to elucidate age and obesity-related deficits. Currently, the literature examining unconventional T-cell response to exercise [7], and especially MAIT-cell response, is quite limited. To date, only acute aerobic exercise in healthy young men [16,17] and acute and chronic exercise in breast cancer survivors [19] have been published. The limitations and strengths of the study are presented to contextualize the findings of the present study. 

### 4.1. Limitations and Strengths

Despite several novel aspects of this preliminary study such as combined acute aerobic and resistance exercise and including both EX and ED groups, there were some important limitations to this study. The current study was a subset of a larger project, with a fixed sample size that left this preliminary analysis underpowered to detect differences in the interaction. As such, we have opted to use effect sizes as the primary comparison between EX and ED. By providing the effect sizes, these data may prove useful in estimating sample sizes necessary to adequately power future investigations, with many potential moderate to large effects that would otherwise be discounted if using traditional probability testing criteria alone (e.g., *p* < 0.05). Second, Lean only provided baseline resting samples at one timepoint, so we were unable to compare the acute exercise response of EX to Lean before and after training. However, their baseline sample provides normative data to compare the baseline values of both OW groups at Pre and Post. Additionally, ED served as an experimental control to account for seasonal differences between Pre and Post training and also provided comparison to EX in response to acute exercise in OW to account for diurnal variation. 

### 4.2. Acute Hemotological Response

Circulating hematological time-course changes were observed in EX in both lymphocyte and neutrophil populations, indicating the acute exercise stimulus was sufficient to mobilize these cells. Immediately following exercise, neutrophils increased by 48% (1.3 × 10^3^/µL cells) and then continued to increase 74% (2 × 10^3^/µL cells) above baseline values following one hour of rest. This sustained response during recovery was expected and compares with previous studies in older adults [21,22,23]. Lymphocytes increased by ~2-fold in EX immediately after exercise following by a small, non-significant decrease at one hour while ED (sitting control) was unchanged. This biphasic response is frequently observed [23] and is influenced by both exercise intensity and duration [24,25]. Increases in hemodynamic shear stress and/or β_2_-adrenergic receptors activation from catecholamines following exercise are responsible for the elevations in leukocyte subpopulations [26], with the rapid decline in circulating levels during recovery being the result of these cells translocating into peripheral tissues such as the gut or lungs [27,28]. 

### 4.3. Circulating Baseline MAIT-Cells

Between groups, medium (0.40–0.54) to large (0.86–3.27) effects were observed when comparing OW and Lean. OW had lower cell counts and frequencies of lymphocytes, MAIT-cells, and MAIT-cell cytokine expression at Pre resting values. Circulating MAIT-cell frequency in OW was 1.1%, which is comparable with other studies investigating overweight and obese populations [14,29] and was 3.9% in Lean which is also comparable with existing literature [30]. Cytokine expression in both OW/Lean was reduced compared to previous studies which examined healthy young males [16,17] or breast cancer survivors and healthy controls [19]. There may be an obesity-related effect on MAIT-cells, as the BMI of participants in the current study was higher (32.9 kg/m^2^) than previous work from our laboratory [19] and is consistent with previous work [7,14,30]. Following exercise training, differences between baseline MAIT-cell % in EX/ED and Lean persisted. While substantial body mass reductions via bariatric surgery previously demonstrated a rescuing effect on MAIT-cells [14], however, in this study baseline resting values were largely unchanged following exercise training. Lymphocyte count and frequency remained reduced in EX/ED compared to Lean. Existing literature investigating the effect of obesity on circulating lymphocytes is conflicting. Some studies report overweight/obese (BMI range from 29.2–35.0 kg/m^2^) subjects have higher total lymphocyte counts or higher CD4^+^ (and reduced CD8^+^) T- Cell subsets [24,31,32] whereas another report reduced CD4^+^ and CD8^+^ lymphocytes in obese (BMI: 38.4 kg/m^2^) compared to non-obese controls [33]. Further investigation into the impact of obesity on immune function is needed. 

### 4.4. Acute Exercise Effect

Typically, MAIT-cells mobilize and egress in a biphasic response following moderate and high intensity aerobic exercise [16,17,19]. However, in this study a different MAIT-cell response was observed in EX, despite a normal lymphocyte and neutrophil mobilization, that may imply an intrinsic deficit within MAIT cells from obese individuals. ED remained seated to serve as an acute non-exercise control, and small changes were observed. In comparison to a recent study using a similar design, non-cancer controls (age: 58, BMI: 27.3 kg/m^2^) demonstrated a 137% increase in MAIT-cell counts and 1.4% increase in MAIT-cell frequency while breast cancer survivors (age: 57 years BMI: 25.8 kg/m^2^) exhibited smaller increases in MAIT-cell counts (+46%) and frequency (+0.7%) [19]. Following acute walking and resistance training, EX did not exhibit biphasic mobilization of MAIT-cells (counts or frequency) at Pre. Additionally, acute exercise did not affect MAIT-cell cytokine expression (TNF-α or IFN-γ). These findings were unexpected, but we can postulate several reasons for the lack of a MAIT-cell response. Low absolute MAIT-cell counts and frequency could potentially reduce the availability of the cells to mobilize following acute exercise due to the exercise modality or intensity. This was the first study to our knowledge to investigate a combined aerobic (walking) and resistance training exercise bout. It is possible that MAIT-cells do not mobilize to the same extent to this mode and/or intensity of exercise as previously demonstrated following intermittent or continuous cycling [16,17,19], although our hematology data (Table 2) argues against this. We hypothesized that MAIT-cell mobilization would be reduce in OW individuals likely from an obesity-related mechanism such as constitutive low-grade inflammation, however more research is needed to elucidate the specific mechanisms involved in reduced MAIT-cell response to acute exercise. 

### 4.5. Exercise Training Effect

The most interesting finding of this study is the change in MAIT-cell count mobilization from Pre to Post. A large significant interaction was observed where OW failed to mobilize at Pre, but following exercise training EX increased mobilization at Post whereas ED remained unchanged. However, this result may be driven by one individual responding to training more than others. Despite this limitation, it appears that the combination of acute exercise and exercise training are both needed to begin seeing improvements in MAIT-cell mobilization. Similar findings were observed in MAIT-cell frequency and egress. At Pre, EX did not mobilize MAIT-cells, but instead experienced a drop below baseline resting values. In the only other exercise training study we are aware of, following 16-weeks of combined aerobic and resistance training MAIT-cell mobilization was attenuated in breast cancer survivors prior to training but showed substantial improvements after training. Specifically, there was a 2-fold increase in MAIT-cell mobilization in breast cancer survivors demonstrating a greater MAIT-cell response after exercise training [19]. In the current study, exercise training appears to improve MAIT-cell mobilization in OW women at Post but not to the same extent as previous work. EX and ED were older and had a higher BMI than the participants in the previous training studies [19], suggesting the possibility of age- and obesity-related deficits. Additionally, Hanson and colleagues [19] used a 16-week intervention, so exercise training duration may also be influencing the greater MAIT-cell rescuing effect demonstrated previously. These results suggest that 12 weeks of exercise training may be sufficient in potentially improving obesity induced attenuation in MAIT-cell mobilization. However, MAIT-cell cytokine response was minimal at Pre and Post thus suggesting exercise training may improve counts without an increase in cytokine production following mitogenic stimulation. 

## 5. Conclusions

Obesity is a major public health concern impacting many facets of health including immunity. MAIT-cells are important unconventional T-cells that possess qualities of innate and adaptive immunity, are numerically low in obesity, have been shown to be increased with bariatric surgery in obese populations, and are responsive to exercise in young healthy individuals as well as older and diseased (cancer) populations. Therefore, we investigated the impact of both acute and chronic combined (aerobic and resistance training) exercise on MAIT-cell counts and frequencies in post-menopausal OW women. Interestingly, MAIT-cells did not respond to acute exercise prior to exercise training, suggesting obesity may be reducing their response. Exercise training may have the potential to partially rescue MAIT-cell response to an acute stressor in post-menopausal OW women. Following training, MAIT-cells demonstrate greater sensitivity to physical stress, which is not seen at baseline. However, future research is needed to better understand the age and disease related declines in MAIT-cell response to exercise. 

## Figures and Tables

**Table 1:** Participant characteristics before (Pre) and after (Post) exercise training.

	Pre					Post			
	OW (n = 16)		Lean (n = 8)	OW vs. Lean		OW (n = 16)		EX vs. ED	
	EX	ED	Lean	MD	ES	EX	ED	MD	ES
Age (y)	61 (3)	65 (4)	64 (7)	1 (4)	0.20	61 (3)	65 (4)	4 (1)	1.13
BMI (kg/m^2^)	33.1 (5)	32.9 (2)	21 (2) #	12 (2)	4.75	33.0 (5)	33.5 (2)	1 (3)	0.13
Height (cm)	164 (4)	161 (6)	161 (5)	2 (0)	0.39	164 (4)	162 (7)	2 (3)	0.35
Weight (kg)	89 (12)	86 (13)	56 (5) #	32 (8)	8.74	89 (12)	88 (13)	1 (1)	0.08
VO_2_ (mL/kg/min)	22 (3)	22 (4)	30 (5) #	8 (2)	2.22	24 (4)	22 (4)	2 (0)	0.50

Reported as mean (SD). At Pre, EX/ED were compared to Lean. At Post, EX and ED were compared after having undergone different interventions. Abbreviations: Pre, pre-training; Post, post-training; EX, exercise group; ED, education group; Lean, lean control group; OW, overweight/obese participants; y, year; kg/m, kilogram/meter; cm, centimeter; kg, kilogram, mL/kg/min, milliliter/kilogram/min; MD, mean difference (absolute change); ES, effect size. # *p* < 0.05 vs. OW.

**Table 2:** Pre-Training Complete Blood Counts in Response to Acute Exercise (Ex) or sitting controls (ED).

Outcome	Group	Base	0 h	1 h	Mob. MD	Mob. ES	Egress MD	Egress ES
Leukocytes (× 10^3^/µL)	EX	4.8 (1.7)	7.3 (2.2) †	6.7 (2.0) †	2.5 (0.4)	5.1	−0.6 (0.1)	2.0
	ED	5.7 (0.9)	6.3 (1.2) †	6.7 (1.3) †	0.6 (.3)		0.4 (0.0)	
Lymphocytes (× 10^3^/µL)	EX	1.5 (0.3)	2.5 (0.6) †	1.3 (0.3)	1.0 (0.3)	3.6	−1.1 (0.3)	5.4
	ED	1.8 (0.5)	1.9 (0.6)	2.1 (0.6)	0.2 (0.1)		0.1 (0.0)	
Neutrophils (× 10^3^/µL)	EX	2.7 (1.3)	4.0 (1.7) †	4.7 (1.8) †	1.2 (0.3)	3.1	0.7 (0.1)	5.0
	ED	3.2 (0.5)	3.6 (0.6) †	3.9 (0.7) †	0.4 (0.2)		0.2 (0.1)	
Hemoglobin (g/dL)	EX	12.9 (1.5)	13.7 (1.5)	13.0 (1.3)	0.8 (0.2)	1.9	−0.7 (0.2)	5.7
	ED	12.9 (1.3)	13.3 (1.3)	13.5 (1.3)	0.5 (0.1)		0.2 (0.1)	
Hematocrit (%)	EX	38.1 (2.5)	40.6 (3.3) †	38.2 (2.2)	2.2 (0.8)	1.2	−2.0 (1.1)	2.9
	ED	39.5 (3.0)	39.2 (3.3)	39.5 (1.9)	1.4 (0.5)		0.3 (1.0)	
PV Shift (Δ%)	EX	-	−9.8 (4.3) †	−0.5 (4.6)	-	-	9.3 (0.4)	24.3
	ED	-	−5.6 (4.8) †	−7.3 (4.3) †	-		−1.7 (0.5)	

Data are mean (SD). Abbreviations: EX, exercise group; ED, education group; Base, baseline; 0 h, immediately post-exercise; 1 h, 1-h post-exercise; Mob., mobilization; PV, plasma volume; MD, mean difference; ES, effect size. † *p* < 0.05 vs. Base.

## Data Availability

Data will be provided upon author contact.
